# Retraction: Hepatic Branch Vagus Nerve Plays a Critical Role in the Recovery of Post-Ischemic Glucose Intolerance and Mediates a Neuroprotective Effect by Hypothalamic Orexin-A

**DOI:** 10.1371/journal.pone.0236818

**Published:** 2020-07-22

**Authors:** The PLOS ONE Editors

After this article [1] was published, the corresponding author requested its retraction due to concerns about results reported in Figure 2 and the outcome of an institutional investigation.

This work was investigated by the Investigation Committee on Misconduct in Research Activities at Kobe Gakuin University, who found that the results reported in the histograms in Figure 2A-F differed substantially from the raw data in a manner that affected the conclusions of the article.

In light of the above concerns, the *PLOS ONE* Editors retract this article.

ST agreed with the retraction. The other authors either could not be reached or did not respond directly to comment on the retraction decision.
